# More Than Resveratrol: New Insights into Stilbene-Based Compounds

**DOI:** 10.3390/biom10081111

**Published:** 2020-07-27

**Authors:** Paulina Pecyna, Joanna Wargula, Marek Murias, Malgorzata Kucinska

**Affiliations:** 1 Department of Genetics and Pharmaceutical Microbiology, University of Medical Sciences, Swiecickiego 4 Street, 60-781 Poznan, Poland; paulinasawicka@ump.edu.pl; 2 Department of Organic Chemistry, University of Medical Sciences, Grunwaldzka 6 Street, 60-780 Poznan, Poland; jkruk@ump.edu.pl; 3 Department of Toxicology, University of Medical Sciences, Dojazd 30 Street, 60-631 Poznan, Poland; marek.murias@ump.edu.pl

**Keywords:** stilbene analogues, isorhapontigenin, pinosylvin, DMU-212, combretastatin, benzanilide derivatives, thiobenzanilides

## Abstract

The concept of a scaffold concerns many aspects at different steps on the drug development path. In medicinal chemistry, the choice of relevant “drug-likeness” scaffold is a starting point for the design of the structure dedicated to specific molecular targets. For many years, the chemical uniqueness of the stilbene structure has inspired scientists from different fields such as chemistry, biology, pharmacy, and medicine. In this review, we present the outstanding potential of the stilbene-based derivatives. Naturally occurring stilbenes, together with powerful synthetic chemistry possibilities, may offer an excellent approach for discovering new structures and identifying their therapeutic targets. With the development of scientific tools, sophisticated equipment, and a better understanding of the disease pathogenesis at the molecular level, the stilbene scaffold has moved innovation in science. This paper mainly focuses on the stilbene-based compounds beyond resveratrol, which are particularly attractive due to their biological activity. Given the “fresh outlook” about different stilbene-based compounds starting from stilbenoids with particular regard to isorhapontigenin and methoxy- and hydroxyl- analogues, the update about the combretastatins, and the very often overlooked and underestimated benzanilide analogues, we present a new story about this remarkable structure.

## 1. Introduction

Medicinal chemistry, as its name denotes, combines the biological and chemical points of view. The main mission of this exciting science is improving the bioactive structure by both: great strides forward of chemical synthesis and our understanding of the biological processes involved in diseases including cancer, neurodegenerative, or metabolic diseases. Based on the statement that a chemical scaffold is defined as the structural core of a chemical compound [1], the concept of scaffold is a starting point in the design and development of new frameworks and gives a new lease of life to well-known structures. There is abundant literature [1,2,3,4,5,6,7,8,9,10,11,12] that presents various chemical frames and highlights the importance of this concept in different disciplines linked with drug discovery such as chemistry, pharmacy, and medicine. Considering a wide range of biological activities and a plethora of possible modifications, the stilbene is one of the most potent scaffolds in medicinal chemistry [2,13,14].

Stilbene is a versatile structure, characterized by two aromatic rings linked by an ethylene moiety. Stilbene exists in two diastereoisomeric forms, E-1,2-diphenylethylene (*trans*-configuration) and Z-1,2-diphenylethylene (*cis*-configuration), while the E isomer is the most common configuration [2]. Natural stilbenes are produced by several plants to protect themselves against stress conditions such as excessive ultraviolet (UV) irradiation, heat exposition, insects’ attacks, and fungus or bacterial infections [15]. Besides the wide variety of natural stilbenes, including hydroxylated [16,17], methoxylated [18,19], glycosylated [20], or prenylated [21] derivatives, the various chemical modifications of natural stilbene have been designed to increase potency, selectivity and improve physicochemical, biochemical, and pharmacokinetic properties and thus, create the huge library of useful structures.

For many years, the stilbene scaffold has been known as an excellent structure in terms of biological potential. To date, both natural and synthetic compounds build on the stilbene scaffold demonstrated a plethora of biological activity such as anticancer [14], anti-inflammatory [22], antimicrobial [23], antifungal [24], and neuroprotective [25] properties and potential agents for diabetes [26] and obesity [27] treatment. Interestingly, in 2006, Li et al. synthesized new stilbene derivatives with substituted hydroxyl groups and found that two of these compounds (E)-2′,3,5′,5-tetrathydroxystilbene and (E)-3′,5,5′,6-tetrahydroxystilbene-2-nitrogen inhibited SARS coronavirus replication using in vitro model [28]. A recent study showed that stilbene-based compounds might also be considered as promising anti-COVID-19 drug candidates acting through disruption of the spike protein [29].

Several stilbene-based drugs are approved for use, e.g., raloxifene, toremifene, or tamoxifen [30]. Furthermore, some structures are under ongoing clinical trials, such as resveratrol (e.g., chemoprevention- NCT04266353, cystic fibrosis-NCT04166396, chronic obstructive pulmonary disease- NCT03819517), combretastatin A1 di-phosphate/CA-1P, also known as OXI-4503 (acute myelogenous leukaemia and myelodysplastic syndromes-NCT02576301), tapinarof, also known as Benvitimod (plaque psoriasis-NCT03956355, NCT03983980, NCT04053387) or pterostilbene (endometrial carcinoma-NCT03671811, acute kidney injury-NCT04342975) which are listed at ClinicalTrials.gov. Moreover, it was found that Ramizol^®^, a first-in-class stilbene-based investigational antibiotic is effective against 100 clinical isolates of Clostridium difficile [31] and currently is under pre-clinical testing for the treatment of *C.difficile* associated disease [32,33]. Notably, the stilbene structure is not only limited to biology and pharmacological science. Stiff-stilbenes (1,1′-diindanylidene), a fuse ring analogue of the stilbene, have been widely explored as molecular rotors, molecular force probes, and optical switches [34]. This group has been regularly used as a model compound in theoretical studies of stilbene photoisomerization [35]. Stiff-stilbenes have drawn the attention of scientists mainly because of their important properties, such as (i) high quantum yield for photochemical isomerization, (ii) the high thermal stability of the Z isomer, (iii) the straightforward synthesis, and (iv) the large geometrical change upon isomerization [35]. All these advantages may be used to develop smart materials or new fluorophores [34,35]. Moreover, it was found that stiff-stilbene may also be used to design selective anticancer and antiparasitic compounds [36]. Stilbene scaffold offers several applications, which may be used by different scientific disciplines. The importance of this scaffold is particularly clear in the light of numerous studies dedicated to this structure. In last year (2019–2020), stilbene as a keyword in PubMed could be found in over 900 papers. Thus, undoubtedly, it is a privileged structural frame of a huge family of bioactive compounds, including natural and synthetic molecules.

**Figure 1:**
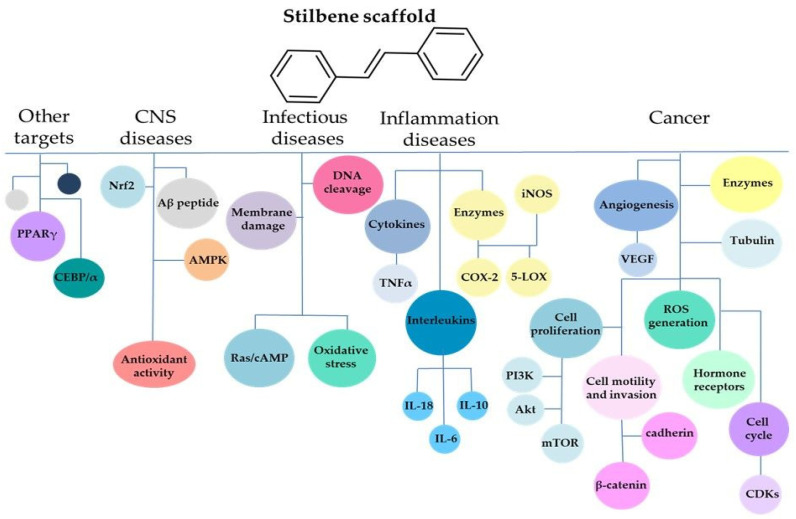
Selected biological activities and specific targets of the stilbene-based compounds. Abbreviations: 5-LOX, lipooxygenase; Akt, protein kinase B; AMPK, AMP-activated protein kinase; CDKs, cyclin-dependent kinases; CEBP/α, CCAAT-enhancer binding protein alpha; COX-2, cyclooxygenase-2; IL, interleukins; iNOS, Inducible nitric oxide synthase; mTOR, mammalian target of rapamycin kinase; Nrf2, nuclear factor erythroid 2–related factor 2; PI3K, phosphoinositide 3-kinases; PPARγ, Peroxisome proliferator-activated receptor gamma; Ras/Camp, cAMP-dependent protein kinase; TNFα, tumor necrosis factor α; VEGF, vascular endothelial growth factor.

In this review, we explore the outstanding potential of the stilbene scaffold (Figure 1). Natural product research, together with powerful possibilities given by synthetic chemistry, may offer an excellent approach for discovering new structures and identifying its therapeutic targets. With the development of scientific tools, sophisticated equipment, and a better understanding of the disease pathogenesis at the molecular level, the stilbene scaffold has inspired innovation in science. This paper mainly focuses on the stilbene-based compounds beyond resveratrol (RSV), which are particularly attractive due to their biological activity. Given the “fresh outlook” about different stilbene-based compounds starting from stilbenoids, with particular regard to isorhapontigenin and methoxy and hydroxyl analogues, through the update about the combretastatins and the very often overlooked and underestimated benzanilide analogues we present the new story about this remarkable structure.

## 2. From Hydroxyl to Methoxy-Stilbene Derivatives

The presence of the hydroxyl group (−OH) may be related to numerous biological effects, such as antioxidant, anticancer, neuroprotective, anti-inflammatory, antibacterial, and antiviral activity [28,37,38,39]. Ashikawa et al. investigated the role of hydroxyl groups in stilbene inhibit TNF-induced NF-κB activation. They found that anti-inflammatory activity is dependent on the presence of hydroxy group and stilbene (lack of hydroxyl groups) and rhaponticin (two hydroxyl groups) had no effect on NF-κB level, while RSV and piceatannol affected NF-κB activation [40]. It should be noted that the hydroxylated but not methoxylated resveratrol derivatives also showed a high rate of COX-2 inhibition [41]. The COX-2 is the principal isoform that participates in the development of inflammation. The ability to inhibit both enzymes constitutive COX-1 and inducible COX-2 were evaluated using in vitro inhibition assays for COX-1 and COX-2 by measuring prostaglandin E2 (PGE2) production and confirmed by quantitative structure–activity relationship (QSAR) analysis and docking studies [41]. Therefore, the stilbene-based compounds with hydroxyl groups are one of the most prominent phenolic hydroxyls that exert a wide variety of biological and pharmacological activities [17,42]. Structure-activity studies have revealed that increasing the number of ‒OH groups at their *ortho* position on the phenol ring of stilbenes could increase the antioxidant [43,44] and cytotoxic activity [45]. However, although hydroxylated stilbene analogues possess great therapeutic potential, its fast metabolism and weak bioavailability may limit their clinical application [14,46]. Therefore, modifying the polyphenolic structures may improve their properties and activity. One of the most common structural modifications observed in aromatic ‒OH groups is their methylation. The O-methylation in stilbene ring increases the lipophilicity to promote cell uptake, protect from degradation, and improve the stability [47]. For example, the presence of two methoxy groups in the pterostilbene structure increases lipophilicity and bioavailability, and due to the presence of only one unhindered hydroxyl group, it is also more metabolically stable [48]. Both hydroxylated and methoxylated stilbene derivatives are fascinating due to their biological activity. Tang et al. reported that the structural modification of stilbene analogues on ring A and ring B strongly affected binding affinities to the proteins and their free radical scavenging activity [44]. It was found that hydroxylation may decrease the affinity, while and methoxylation enhances the affinity to the protein [44]. Thus, the number and position of methoxy and hydroxyl groups should be carefully balanced.

**Figure 2:**
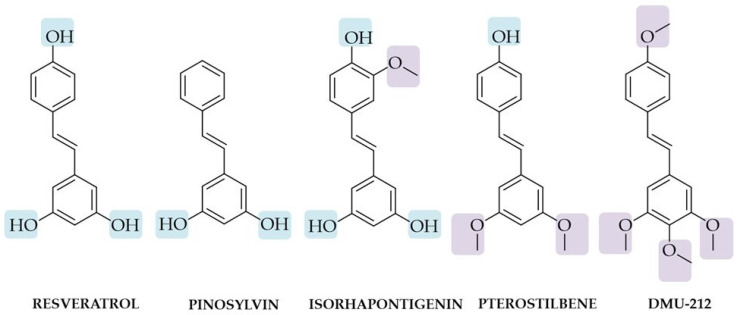
The chemical structures of resveratrol, pinosylvin, isorhapontigenin, pterostilbene, and DMU-212.

To date, different hydroxyl- and methoxy- stilbene derivatives have been synthesized and characterized. In this chapter, the three of selected compounds belong to a different class of stilbene analogues such as *trans*-3,5,4′-trihydroxy-3′-methoxystilbene-isoprhapontigenin (hydroxylated and methoxylated stilbene analogue), 3,5-dihydroxystilbene-pinosylvin (hydroxylated stilbene analogue), and *trans*-3,4,5,4′-tetramethoxystilbene, also known as DMU-212 (methoxylated RSV analogue) will be described to show their interesting activity (Figure 2).

### 2.1. Isorhapontigenin—Successor of Resveratrol

Isorhapontigenin (*trans*-3,5,4′-trihydroxy-3′-methoxystilbene, ISO) is a resveratrol analogue with methoxy groups at the ring B, found in the Chinese herb *Gnetum cleistostachyum* and grapes [49]. Dai et al. characterized the pharmacokinetic profile of ISO and RSV in Sprague-Dawley rats after a single oral administration (200 µmol/kg b.w.) [50]. Interestingly, both ISO and RSV were absorbed rapidly, while better oral pharmacokinetic profile was observed for ISO, and bioavailability was two to three folds greater than that of RSV [50]. These data confirmed that from the pharmacokinetic point of view, ISO is a better candidate for drug development than resveratrol [50]. It is more important because the low oral bioavailability of RSV (<1%) due to rapid and extensive metabolism in the intestine and the liver is one of the most drawbacks of this compound [51]. The favourable pharmacokinetic profile was also observed by Yeo et al., where oral bioavailability was two-fold higher than RSV [52].

ISO is well-known for its various beneficial effects, including antiplatelet activity [49]. Comparing to RSV, it exhibited a more substantial selective inhibitory effect on ADP-induced platelet aggregation. Furthermore, Lu et al. investigated that ISO may act as a reactive nitrogen species (RNS) scavenger, which confirms the antioxidant potency of this stilbenoid [53]. Furthermore, theoretical studies showed that ISO is also an effective scavenger of hydroxyl (OH^●^) and hydroperoxyl (HOO^●^) radicals [54]. Other studies, including the research with 19 stilbenoids, on human monocytic THP-1 cell line (THP1-XBlue™-MD2-CD14) and human hepatoma cell line (HepG2), showed that ISO has mild antioxidant activity compared to piceatannol. Interestingly, ISO activated the increased expression of the nuclear factor erythroid 2-related factor 2 (Nrf2), the transcription factor that controls the expression of genes encoding cytoprotective enzymes and protein [55].

The anti-inflammatory activity of ISO was observed in the study conducted on rats and cell culture of chondrocytes. The effect was obtained throughout suppression of interleukin-1β inhibit the nitric oxide (NO), inducible nitric oxide synthase (iNOS), PGE2, and cyclooxygenase-2 (COX-2) [56]. ISO also exerts better anti-inflammatory activity compared to RSV [52]. Another study performed on patient-derived human airway epithelial cells from healthy subjects and patients with chronic obstructive pulmonary disease (COPD), showed that ISO suppressed the PI3K/Akt pathway. A recent study showed that activation of the PI3K/Akt signalling pathway might be involved in steroid resistance in COPD [57]; thus, ISO can be considered as a potential drug for corticosteroid-resistant COPD.

It has been reported that ISO exerts the anti-diabetic activity confirmed by in vivo study [26]. ISO treatment among mice at the dose of 25 mg/kg b.w. or vehicle intraperitoneally for five weeks significantly reduced three markers of diabetes: postprandial levels of glucose, insulin, and free fatty acids. As a result, changes in adipose tissue reduction were observed, and adipose insulin sensitivity was also improved. What is more, the mRNA expression levels of PPARγ (peroxisome proliferator-activator receptor gamma) were higher in white adipose tissue among mice treated by ISO comparing to the control group [26].

Other study showed that one-week daily oral administration of ISO at a dose of 25.8 mg/kg b.w., affected the glucose and plasma cholesterol level, fatty acid biosynthesis, and amino acid/arachidonic acid metabolism. That indicates the health-promoting role of this compound [50].

ISO has recently been identified as a promising anticancer agent. Fang et al. showed that ISO might decrease bladder cancer T24T cell line viability with an IC_50_ value of 55.2 ± 2.3µM [58]. Moreover, ISO exerted anticancer effect by apoptosis induction in human bladder cancer T24T, UMUC3, RT112 cell line, and colon cancer HCT116 cell line. It was found that ISO induced apoptosis via downregulation of X-linked inhibitor of apoptosis protein (XIAP) by inhibition of Sp1 (specificity protein 1) expression, transactivation, and the binding activity of Sp1 to the *xiap* promoter in T24T (at a dose of 60µM) [58]. Further in vivo study confirmed that ISO inhibited tumour growth in T24T-tumour-bearing xenograft mice. Treating mice with ISO at a dose of 150 mg/kg b.w. for six weeks reduced tumour mass, which was associated with the downregulation of Sp1 and Cyclin D1 expression in cancer tissue [59]. Furthermore, Xu et al. showed that ISO at concentration range 10–80 µM downregulates cyclin D1 and SOX2 as a result of the induction of miR-145 in patient-derived glioblastoma spheres (PDGS) [60]. Another study showed that ISO at concentrations 10µM and 20µM suppressed the growth of human bladder cancer T24T and UMUC3 cell lines in vitro by upregulation of the forkhead box class O 1 (FOXO1) mRNA transcription. Targeting the FOXO1 activity by ISO treatment resulted in the downregulation of matrix metalloproteinases-2 (MMP-2) and inhibited cancer cell invasion [61]. These authors also demonstrated using C57BL/6J male mice that ISO (150 mg/kg/day in drinking water for 20 weeks) might inhibit mouse-invasive cancer growth induced by N-butyl-N-(4-hydroxybutyl) nitrosamine (BBN) (0.05% in drinking water for 20 weeks) [61]. Luo et al. proved that ISO at a concentration of 20 μM used in T24T cell culture, could affect CD44 protein and *cd44* mRNA expression through decreases in Sp1 direct binding, which inhibited stem cell-like phenotypes and invasiveness of the tumour [62]. It has also been observed that protein expression involved in the DNA damage pathway, ubiquitin-specific peptidase 28 (USP28) was reduced; as a result, CD44 protein stability was decreased [62].

Furthermore, ISO causes the induction of miR-4295, which inhibited the *usp28* translation and expression in bladder cancer cells [62]. Interestingly, ISO also exerts an antiproliferative effect on both estrogen-dependent human cancer cell lines MCF-7 (the IC_50_ value of 34.16 µM) and T47D, and triple-negative breast cancer cell line MDA-MB-231 by induction of oxidative stress and cell cycle arrest [63]. Another study showed anticancer activity of ISO against prostate cancer LNCaP and CWR22Rv1 cell lines. It should be emphasized that ISO did not affect cell viability against benign hyperplasia epithelial cells (BPH-1) and healthy prostate cells (WPMY-1) [64]. It was shown using MTT assay that ISO inhibited the viability of LNCaP and CWR22Rv1 in a dose-dependent manner. ISO at a concentration of 100 µM decreased prostate cancer LNCaP and CWR22Rv1 cells viability to 25% and 45%, respectively. On the other hand, the weak effect was exerted on BPH-1 and WPHY-1 cells, where cell viability was ~80% after ISO treatment at the same treatment conditions [64]. This study showed that ISO might bind to epidermal growth factor receptor (EGFR) and inhibit its autophosphorylation and downstream signallings, such as PI3K/Akt and extracellular signal-regulated kinase 1/2 (ERK1/2) pathway. Moreover, as a result of inhibition of FOXO1 phosphorylation, the FOXO1 is translocated to the nucleus, thus resulted in the activation of several pro-apoptotic proteins. ISO could also decrease androgen receptor (AR) protein level and downregulated AR activity by two mechanisms: (i) reducing the activity of Sp1 and (ii) promoting the ubiquitination/degradation levels of AR proteins. The efficacy of ISO was also demonstrated in the tumour xenograft model using CWR22RvI cells. Mice were treated with ISO (at a dose of 5 mg/kg b.w.) every two days for one month. Compared to control mice, the results from the ISO-treated group indicated the important role of EGFR- related pathways in cancer growth inhibition and apoptosis [64].

Zakova et al. also demonstrated antimicrobial activity of ISO [65]. They proved that ISO inhibited eight strains of *Staphylococcus aureus* with MIC (minimal inhibitory concentrations) ranging from 128 to 256 µg/mL. The study was conducted using six reference strains obtained from ATCC (American Type Culture Collection) and two clinical samples. It should be noted that RSV did not show such a strong antimicrobial activity as ISO [65].

The summary of biological activity and molecular effects of ISO is presented in Table 1.

### 2.2. Pinosylvin—Stilbene of Underestimated Importance

The following compound, pinosylvin (3,5-dihydroxy-*trans*-stilbene), is the natural polyphenol, *trans*-stilbenoid found in heartwoods and leaves of *Pinus sylvestris* that possess numerous biological properties [72]. It is produced by plants as a secondary metabolite to protect against insects and microbes [79]. Pinosylvin exerts various biological activities, which are shown in Table 1.

The anti-inflammatory effect was observed using in vitro and in vivo models by Eräsalo et al. [67]. Pinosylvin at a dose of 3 μM, suppressed PI3K/Akt pathway in stimulated macrophages (J774 cell line). Interestingly, from all tested stilbenoids, the better effect was only observed for monomethylpinosylvin [67]. Furthermore, the nitric oxide (NO) production was restrained at a concentration of 16.6 µM. Pinosylvin decreased by 50% the expression of two cytokines: interleukin 6 (IL-6) and monocyte chemoattractant protein 1 (MCP1) at a dose of 32.1 µM and 38.7 µM, respectively. Moreover, the anti-inflammatory effect was observed in vivo when mice were treated with a single dose of 30 mg/kg b.w. [67]. Laavaola et al. also found that pinosylvin decreased iNOS expression (EC_50_ value of 12 µM), decreased NO production with an EC_50_ value of 13 µM, and reduced the production of IL-6 (at a dose of 30 µM) in J774 cells [68]. In human osteoarthritis chondrocytes (OA), pinosylvin reduced the IL-6 expression level and protein of both IL-1β and IL-17. Notably, NF-κB was inhibited at a dose of 100 µM in human T/C28a2 chondrocytes [68] The anti-inflammatory activity was confirmed in vivo, where pinosylvin at the dose of 100 mg/kg b.w. decreased oedema in C57BL/6 mice [69].

The pinosylvin influence was also observed in transient receptor potential ankyrin 1 (TRPA1), which plays an important role in sensory neurons. The TRPA1- mediated Ca^2+^ influx was inhibited (IC_50_ of 26.5 µM), which reduced the inflammatory process. It has also been confirmed in the in vivo study conducted on male mice (IC_50_ of 16.7 µM), that pinosylvin reversed the effect of TRPA1 agonist. Curiously, a high concentration (a dose of 100 µM) showed a minor activating effect TRPA1; the same was seen after RSV administration (a dose of 100 µM). Moilanen et al. confirmed the anti-inflammatory effect; the pinosylvin administration also blunted IL-6 to the same mice [66].

Other studies also confirmed interesting properties of this stilbene, such as antidiabetic and anticancer activity. Rat L6 myoblasts were incubated with pinosylvin enhanced basal glucose uptake, at all tested concentrations (20–100 µM) [70]. However, the best effect was observed at the dose 60 µM, while at higher concentration (100 µM), insulin-stimulated glucose uptake was inhibited. The glucose transporter 4 (GLUT4) translocation was observed only in basal conditions, at the dose of 60 µM and 100 µM [70]. Moreover, pinosylvin, at a dose of 100 µM, activated sirtuin-1 (SIRT1) with an EC_50_ value of 116.8 ± 7.5 μM. Furthermore, the increased AMP-activated protein kinase (AMPK) phosphorylation was also observed [70]. Modi et al. also demonstrated that pinosylvin has a beneficial effect on adipocytes [71]. Treatment 3T3-L1 adipocytes cells with pinosylvin at a concentration of 60 µM for ten days inhibited cells proliferation. Furthermore, two important regulators of adipogenesis, PPARγ and C/EBPα, were also down-regulated. Interestingly, the reduction of both regulators was observed at the same level as resveratrol (60 µM). The reduction of IL-6 secretion was observed in pinosylvin-treated cells [71].

It was found that leukaemia cells (THP-1 and U937) treated with pinosylvin induced both apoptotic and autophagic cell death pathways [73]. Pinosylvin at a dose of 100 µM, promoted caspase-3 activation, accumulation of a standard marker for autophagosomes (LC3-II), and the down-regulation of AMP-activated protein kinase α1 (AMPKα1) [73]. On the other hand, the study conducted on tongue squamous carcinoma cells (SCC9, SAS, and HSC-3 cell lines) confirmed that pinosylvin reduces the phosphorylation of ERK1. As a result, migration and invasion were restrained [72].

### 2.3. Short Story about DMU-212—When Synthetic Chemistry Achieved a Success

The substitution of the hydroxyl group with the methoxy group would be a clear step toward improving its pharmacokinetic and pharmacodynamic properties. As mentioned, RSV has low bioavailability due to being metabolized by sulfation and glucuronidation in the liver. Bioavailability can be increased by modification of the hydroxyl group, and its methylation may prevent the fast metabolism and increase the lipophilicity. The fully methylated analogue of resveratrol, (E)-3,5,4′-trimethoxystilbene (TMS) may act as a vascular-targeting agent [80]. Moreover, Traversi et al. found that structural isomer (Z)-TMS, exerted a strong anti-proliferative activity (100-fold more active than RSV) and caused cell cycle arrest at the G2/M phase and inhibition of tubulin polymerization [81]. Furthermore, the stilbenoid analogue with another methoxy group at position -4′, (E)-3,4,5,4′-tetramethoxystilbene, also known as DMU-212, possesses enhanced anticancer activity in terms of the induction of apoptosis, inhibition of cell growth compared to RSV. The anticancer mechanism and potential molecular targets of DMU-212 are described in Table 1. In 2004, Sale at al. carried out the first study concerned the DMU-212 metabolism and showed that, contrary to RSV, DMU-212 underwent metabolic hydroxylation or single and double O-demethylation [82]. The levels of DMU-212 in the brain, small intestinal, and colonic mucosae after DMU-212 administration exceeded levels of RSV [82]. Furthermore, these results suggested that DMU-212 is capable of crossing the blood-brain barrier more easily than RSV due to the higher lipophilicity. The intensive study performed by Androutsopoulos et al. showed that DMU-212 is metabolized in vivo to four major metabolites: (E)-3′-hydroxy-3,4,5,4′-tetramethoxystilbene (DMU-214), (E)-4′-hydroxy-3,4,5-trimethoxystilbene or (DMU-281), (E)-4-hydroxy-3,5,4′-trimethoxystilbene (DMU-291), and (E)-3-hydroxy-4,5,4′-trimethoxystilbene (DMU-807) [83]. The authors also observed the strongest anti-proliferative activity of DMU-214, among the other metabolites of DMU-212 in the breast (MCF-7) and liver (HepG2) cancer cells [83]. The further studies showed that DMU-212 metabolites exerted the anti-proliferative (at a concentration range of 0–1 μM) and pro-apoptotic (at a concentration of 0.125 µM and 0.250 μM) effects in ovarian cancer cells (A-2780 and SKOV-3), with a prominent activity, was noticed for DMU-214 against A-2780 cell line [84].

In 2005, McErlane et al. suggested that DMU-212 is inactive until it undergoes aromatic hydroxylation (by CYP1A1) and O-demethylation (by CYP1B1) to generate two active metabolites, DMU-214 (referred as a tyrosine kinase inhibitor) and DMU-291 (apoptosis inducer), respectively [85]. Further studies showed that the human cytochrome P450 1A1 (CYP1A1) is mainly involved in the DMU-212 metabolic pathway [75,86]. The dose-dependent induction of CYP1A1 and CYP1A2 mRNAs was noted after treatment of primary human hepatocytes cells with DMU-212 [75]. This metabolic activation is probably involved in anticancer activity, while it was found that the lack of the expression of CYP1A1 is associated with lower activity of DMU-212 [86]. The important role in CYP1A1 activation may play the aryl hydrocarbon receptor (AhR). In general, AhR regulates the expression of numerous cytochrome CYP450 genes, including members of the CYP1 family, CYP1A1, and CYP1A2 [67]. It was found that DMU-212 induced AhR activation in HepG2 stably transfected line AZ-AhR [75]. In contrast, another study showed that DMU-214 at a concentration of 0.25 µM might decrease the level of the AhR nuclear fraction and thus, exert the inhibitory effect on CYP1A1 [86].

Interestingly, in 2009 Chun at al. showed that 2,2′,4,6′-tetramethoxystilbene is a potent inhibitor of human cytochrome P450 1B1 (CYP1B1), and this property was also shown for DMU-212 [87]. It is particularly important because CYP1B1 is overexpressed in a variety of cancers, such as prostate [88], breast, ovarian [89], colon and bladder cancer [90]. CYP1B1 is involved in the metabolic activation of many environmental pro-carcinogens, as well in the metabolism of endogenous hormones [91,92]. Moreover, the oxidation of anticancer drugs catalyzed by CYP1B1 may be caused resistance to the therapy [93,94,95]. Therefore, the regulation of CYP1B1 expression can act as a therapeutic strategy, especially for cancer treatment. Taking into account the role of CYP1A1 in DMU-212 activation, it is suggested that its metabolite DMU-214 may act as both a CYP1A1 and CYP1B1 inhibitor [86].

Miao et al. demonstrated that DMU-212 induced apoptosis and anti-angiogenesis in cultured human umbilical VECs (HUVECs) after 6 h exposure to 20 µM of this compound [74]. Furthermore, it was found that among 56 altered genes encoding apoptosis, mitogen-activated protein kinase (MAPK) pathway, different enzymes, protein transport, angiogenesis and migration, and cytokines production, 44 of them were up-regulated, and 12 were down-regulated. Moreover, seven randomly selected genes expression (*IL8*, *EGR1*, *ERRFI1*, *TRPC4*, *BIRC3*, *CYP1B1*, and *MVK*) were evaluated using qRT-PCR (quantitative real-time PCR). Obtained results showed a statistically higher expression among five genes (*IL8*, *EGR1*, *ERRFI1*, *TRPC4*, and *BIRC3*), which are responsible for cytokine production, angiogenesis, migration, apoptosis, and protein transport [74]. Moreover, using HUVECs, the increased apoptosis (reduction of Bcl-2, increased caspase-3, and -9) and cell viability inhibition by DMU-212 were observed. A significant reduction of vascular endothelial growth factor (VEGF)-stimulation was also noticed when IC_50_ was 20 μM after 48 h of exposure. Furthermore, a study conducted in vivo on the developing embryos of chicks showed a strong anti-angiogenic effect of the tested compound. Results confirmed that DMU-212 is a potent VEGFR-2 but not VEGFR-1 tyrosine kinase inhibitor [76].

Ma et al. demonstrated that DMU-212 exerts higher growth inhibition in breast cancer cell lines: MCF-7 and MDA-MB-435 by block cell cycle at G2/M phase, decrease Cyclin D1 expression and inhibition signal transducer and activator of transcription 3 (STAT3) phosphorylation, which may impact tubulin polymerization [96]. This result was confirmed in vivo by Cichocki et al., who found that DMU-212 at a dose of 50 mg/kg b.w., decreased the STAT3 activation [97]. Moreover, the authors showed that DMU-212 reduced the pro-inflammatory transcription factors, particularly NF-κB, and as a consequence, iNOS expression [97]. Other studies conducted on human melanoma cells (A375, MeWo, M5, and Bro) using DMU-212 indicated inhibition of proliferation cells (IC_50_ value of 0.5 µM for A375 and Bro; an IC_50_ value of 1.25 µM for MeWo and M5 cells) after 96 h of treatment [77]. The mechanism of activity was the result of mitotic arrest (G2/M), induction of apoptosis, and activation of ERK1/2 protein [77].

Fan et al. reported the effect of DMU-212 and its isomer (Z)-3,4,5,4′-tetramethoxystilbene, on lung cancer cells (H1975, H820, A549, H358) with different EGFR genetic mutations and one healthy lung epithelial cell line (BEAS-2B) [98]. The activity of DMU-212 was lower toward all cell lines (IC_50_ was >40 µM), compared to (Z)-TMS (IC_50_ was 57.2 nM) [80]. The authors also found that the (Z)-TMS inhibited the phosphorylation and activation of EGFR in gefitinib-resistance lung cancer cells, induced caspase-independent apoptosis, and autophagy, and causing endoplasmic reticulum (ER) stress and AMPK activation [98].

It was found that DMU-212 may also impact the antioxidative enzymes in vivo. The rats treated with DMU-212 at the dose of 50 mg/ kg b.w. showed the reduction of superoxide dismutase (SOD-2), catalase (CAT), glutathione peroxidase (GPx), and glutathione reductase (GR), compared to the control group. Furthermore, the administration of DMU-212 at the same dose showed the statistically significant reduction of genes triggering mitochondria-mediated apoptosis in rat liver, such as *apaf*-1, *pten*, and *stat-1*. Furthermore, rats with DMU-212 administration were characterized by higher expression of caspase-9 mRNA [78].

## 3. Combretastatins—Between Bench and Bedside

The combretastatins are a group of diaryl stilbenoid, natural compounds found in the South African willow tree *Combretum caffrum* [99]. From all the isolated, natural combretastatins, two *cis* stilbenes have gained particular attention over the years: combretastatin A-4 (CA-4) and combretastatin A-1 (CA-1). Combretastatins are antimitotic agents that inhibit tubulin polymerization by sharing some structural similarities with colchicine [19,100]. Furthermore, combretastatins were found as vascular targeting agents or vascular disruptors [101,102]. Although combretastatin-based compounds exert prominent anticancer activity, some disadvantages, such as (i) the isomerization of its stilbene Z-double bond into the less active E-form during storage, administration, and metabolism; (ii) low water solubility, and (iii) the non-selective targeting, may limit the transfer to clinics [103,104]. Starting from combretastatin A-4 phosphate (CA-4P, fosbretabulin), more water-soluble update version of CA-4, medicinal chemistry offers further advanced derivatives and formulation such as theranostic nanocarriers [105,106], nanodrugs [107], hypoxia-activated prodrugs [108,109], and others. The remarkable potential of combretastatin analogues is borne out by the fact that currently, CA-4P is studied under several clinical trials as a monotherapy, and combining therapy with other chemotherapeutic agents, such as pazopanib, paclitaxel, carboplatin, or bevacizumab [103]. Each year, numerous excellent works about combretastatins are published to get more insight into chemical modifications and modes of action, based on the success of this molecule. It is important to underline that, nowadays, 40 publications with “combretastatin” as a keyword could be found in PubMed, which were published in the current year.

**Figure 3:**
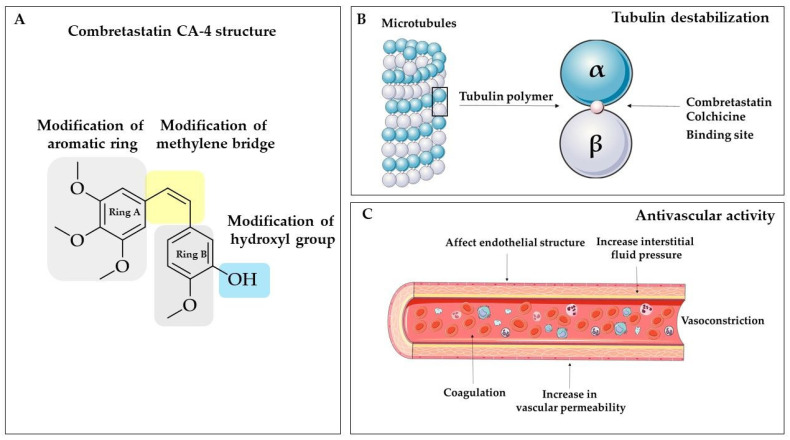
The structure of CA-4 with marked sides of the most useful structural modification (**A**). The anticancer activity of CA-4 associated with disruption of microtubule dynamic and vascular effects are presented in panels (**B**) and (**C**), respectively.

In this chapter, we present a strictly chemical approach to the preparation of new stilbene analogues based on combretastatin as potential drugs. Looking at the combretastatin molecule (Figure 3), it might not appear to offer researchers a wide range of possibilities. However, it turns out that each of the structural elements described is responsible for biological activity. The authors decided to keep the trimethoxybenzene ring as an unchanging fundamental element of the structure. It is vital for tubulin binding and binding at the colchicine binding site of the microtubule [110]. Structure−activity relationship (SAR) studies showed that features, such as (i) *cis*-orientation of both the aromatic rings, (ii) the 3,4,5-trimethoxy moiety on ring A, and (iii) the para-methoxy moiety present on ring B, are important for CA-4 cytotoxic activity [111]. It was found that several antimitotic agents, such as combretastatin A-4, colchicine, steganacin, and podophyllotoxin, may bind at the colchicine site on tubulin due to the presence of trimethoxy aryl unit [112]. Thus, the A ring determines the biological activity of combretastatins, and it is important to exert anticancer activity [19].

### 3.1. Modification of the Aromatic Ring

**Figure 4:**
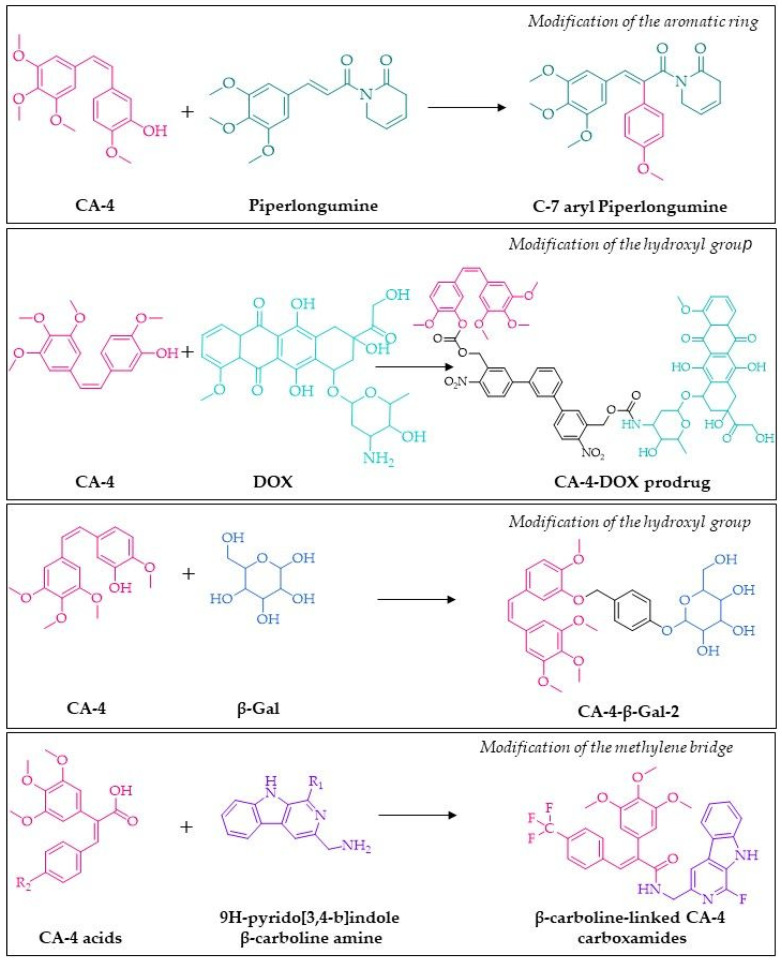
The scheme presented the structure of hybrid compounds, which possess both CA-4 moiety and the additional active moiety [115,116,119,120]. This graph presents the general overview about combretastatin A4-basedh and does not present the detailed synthesis.

One of the strategies for developing new derivatives is to change aromatic rings. In the case of the combretastatin molecule, hybrid techniques are often used, and this approach enables the obtaining of derivatives with prominent biological activities. Currently, the use of hybrid molecules that may simultaneously impact with two or even more targets in cancer cells is more favourable. In general, this approach is based on (i) merging the structural features of different drugs, (ii) conjugating two drugs or pharmacophores via cleavable/non-cleavable linkers [113]. To date, several hybrids linking combretastatin with other active molecules such as celecoxib pharmacophore [114], β-carboline [115], camptothecin [106], and cisplatin have synthesized as promising anticancer agents. Following this strategy, Punganuru et al. designed the piperlongumine (PL) derivatives with an aryl group inserted at the C-7 position [116]. This modification creates a combretastatin A4-like structure, while it does not affect the PL configuration simultaneously (Figure 4). Piperlongumine (also known as piplartine) is a small molecule alkaloid presents in black pepper (*Piper longum*) that is receiving increased interest due to its anticancer activity [117,118]. Basak et al. proposed that reactive oxygen species (ROS) generation by PL and the thiol conjugations may decrease the cellular glutathione (GSH) level and promote protein thiolation [117]. In effect, the glutathionylation of mutant p53 protein may induce structural perturbations in the defective DNA-binding domain of the tumour suppressor and restore some functionality of p53 in cells [117]. Therefore, the combination of CA-4 properties and PL activity may lead to compounds that affect cancer cell growth in vitro by both tubulin polymerization and p53 reactivation [116]. The 4-methoxy substituted C-7 aryl piperlongumine derivative known as KSS-9 exerted a significant anti-tubulin activity and restoration of mutant p53 by increasing wild-type-like protein (pAb1620-reactive) and decreasing in pAb240-reactive mutant protein at the same time [116]. Further insight into the mechanism of action showed that this hybrid increased expression of mouse double minute 2 (MDM2), p21cip1, and p53-upregulated modulator of apoptosis (PUMA), block cell cycle at phase G2/M, increased level of cleaved caspase-3 and cleaved PARP (poly ADP-ribose polymerase) and induced apoptosis [116].

Many reports show the impact of new scaffolds on biological activity, where the activity of one- and multi-cyclic, five-, or six-carbon compounds is discussed [121]. It is well-known that it is critical for the biological activity of combretastatin that there is a presence of *cis* configuration on the methylene bridge. It seems that the introduction of the azaheterocyclic system or scaffold with the methoxy group increases dissolution in water and the potential for tubulin polymerization. Compounds with the thiophene system seem to have a lower effect on tubulin.

In the case of bicyclic systems, the location of the nitrogen atom is crucial [121]. This atom should be on a ring not attached directly to the unsaturated bond. Such derivatives have better solubility in water than the analogues with carbon ring [121]. Similar conclusions can be drawn from the structure−activity relationship (SAR) analysis for indole derivatives and several other aromatic groups [121]. The more interesting bicyclic compounds include benzoxazole derivatives [122]. For many years, the benzoxazole ring has been used in the design of biologically active molecules with the broad spectrum of biological activity, such as antiviral [123], antifungal [122], antibacterial [122] anticancer [122,124,125], and other activities [126]. The (Z)-3-methyl-6-(3,4,5-trimethoxystyryl)-2(3H)-benzoxazolone derivative exerted activity against colon cancer (HT-29), hepatocellular carcinoma (HepG2), erythroleukemic (K562), transformed human umbilical vein endothelial (EA.hy926) cell line and the ability to block a cell cycle in G2/M phase [127]. Moreover, Kumar et al. designed a series of benzoxazole linked combretastatin derivatives with strong anticancer activity against breast (MCF-7), lung (A549), and melanoma (A375) cancer cell lines with IC_50_ values ranging between 0.11 ± 0.093 µM and 17.3 ± 1.33 µM [128].

Naphthalene analogues represent another interesting group of derivatives. Magiure et al. synthesized two agents: 5-hydroxy-6-methoxy-1-aryldihydronaphthalene analogue (KGP03) and 1-aroyldihydronaphthalene analogue (KGP413), and their phosphate prodrug salts were prepared (KGP152 and KGP04) to increase the solubility in water [129]. Both prodrugs exerted cytotoxic activity against NCI-H460 (non-small cell lung), DU-145 (prostate), and SKOV-3 (ovarian) human cancer cell lines [129]. The in vivo studies confirmed their activity to tumour vasculature. KGP152 at a dose of 200 mg/kg b.w. reduced tumour blood flow after 4h in SCID-BALB/c mice bearing MDA-MB-231 tumour. In a different study, KGP04 at a dose of 15 mg/kg b.w. after 2h of incubation, caused a vascular disruption in a Fischer rat bearing an A549 tumour [129]. In further studies, the authors prepared a group of cyclic chalcones and related analogues that incorporate structural motifs of CA-4 and demonstrated their cytotoxic effects against NCI-H460, DU-145, and SK-OV-3 cell lines. Although compounds inhibited cancer cell proliferation, these molecules proved inactive as inhibitors of tubulin polymerization [130].

Of course, these compounds may have a much more complicated structure, such as spiro moiety. Brand et al. has proved that derivatives of the β-nitrostyrene, spiroisatin-dihydroquinoline, pyroisatin-thiazolidinone, or spiroisatin-nitropyrrolizidine type have anticancer (HeLa and Jurkat cell lines), antifungal (*Trychopython mentagrophytes*), or antiviral (Human herpes viruses: HHV-1, HHV-2) properties [131].

The Masked Polar Group Incorporation (MPGI) method is a new strategy to increase molecular polarity without compromising polar groups by incorporating f.e. azaheterocyclic scaffolds. For example, the *ortho* substituents of the pyridine nitrogen in combretastatin analogues hamper it from the hydrophobic molecular target pocket and increase molecular polarity [132]. It has been used by González et al. to improve solubility and increase the activity of the new derivatives [132]. These compounds were found to have better cytotoxic activity but moderate ability to inhibit tubulin polymerization. Their mechanism of action was based on inhibition of the cell cycle at a G2/M phase and induction of apoptosis [132].

In summary, the possibilities of changing the 2-hydroxy-3-methoxy-phenyl ring are enormous and have already been thoroughly investigated by researchers. When modifying the combretastatin molecule by inserting five-membered, six-membered or fused rings, the presence of additional heteroatoms (e.g., nitrogen and oxygen) and substituents (e.g., methoxy and amino) should be taken into account. They will play an essential role in binding to tubulin, and they will significantly modify the solubility of the new derivative in water.

### 3.2. Modifications of the Hydroxyl Group of Combretastatin Core

Etherification, esterification, and substitution may be the most basic methods of modifying this group. For example, the conversion of a hydroxyl group into an ether bond has been used by Doura et al. to create innovative prodrugs for ovarian cancer therapy [120]. A molecule glycoside was hydrolyzed by galactosidase, an enzyme strongly induced in cancer cells. The molecule itself showed better anti-tumour properties, which was confirmed on cell lines (Figure 4). That gives opportunities in the design of platforms that deliver drug molecules to cancer cells [120]. Huang et al. also used the idea of using gluco-conjugates to improve the effectiveness of the drug [133]. The authors proved that conjugates of CA-4 with glucose, mannose, and galactose have improved water solubility and a better safety profile with the 16-34-fold increased maximum tolerated dose values compared to CA-4 [133].

**Figure 5:**
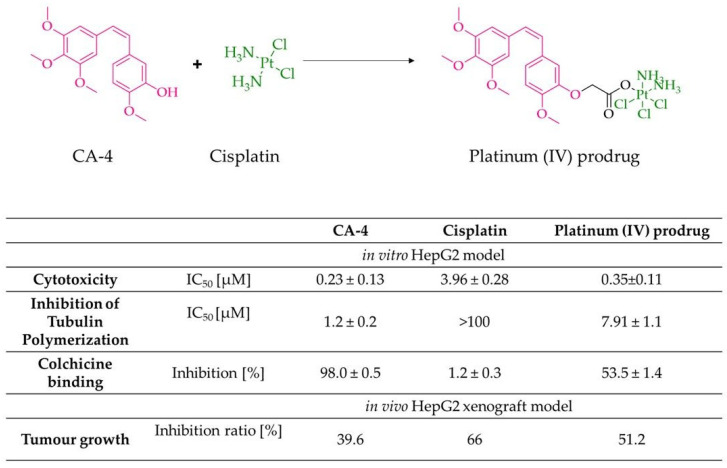
The structure and activity of CA-4, cisplatin, and CA-4-cisplatin prodrug against HepG2 cell line in vitro and in vivo [134]. This graph presents the general overview about combretastatin A4-based hybrid and does not present the detailed synthesis.

An interesting pharmacotherapeutic strategy seems to be combining two drug molecules to obtain a derivative with better biological activity, as mentioned above. Combining combretastatin and camptothecin molecules by an ester moiety can be an example of such a structure. This molecule can form micelles, which may improve solubility with higher cytotoxicity compared to the original drugs [106]. The combination with platinum derivatives also perfectly fits into the above strategy (Figure 5) [134]. In addition to increasing anti-tumour activity, the ability to inhibit tubulin polymerization has been demonstrated. This hybrid inhibited the cell cycle and induced apoptosis in HepG2 cells [134]. Moreover, the anticancer activity was also confirmed in vivo using the HepG2 tumour xenograft model. The tested compound used at a dose of 5 and 10 mg/kg b.w. (administrated intravenously, once a week for three weeks) significantly suppressed tumour growth compared to CA-4 and exhibited low toxicity compared to cisplatin [134].

Furthermore, Liu at al. designed the photoresponsive hybrid prodrug bearing both doxorubicin (DOX) and CA-4 structure (Figure 4) [119]. They found that sequential irradiation at 405 nm and 365 nm led to the release of DOX and CA-4, respectively [119]. The strictly controlled drug releases, which exert different mechanisms of action, might be most beneficial in terms of achieving a synergistic effect by different drugs. To date, it is known that CA-4P may sensitize drug-resistant human breast cancer MCF-7/ADR cells to DOX [135]. Additionally, Zhu et al. showed that the polymersomes dual loaded with CA-4P and DOX could inhibit Pgp function by downregulating protein kinase C alpha (PKCα), stimulating ATPase activity, decrease ATP level and increase the generation of ROS, thus overcoming DOX resistance [135].

**Figure 6:**
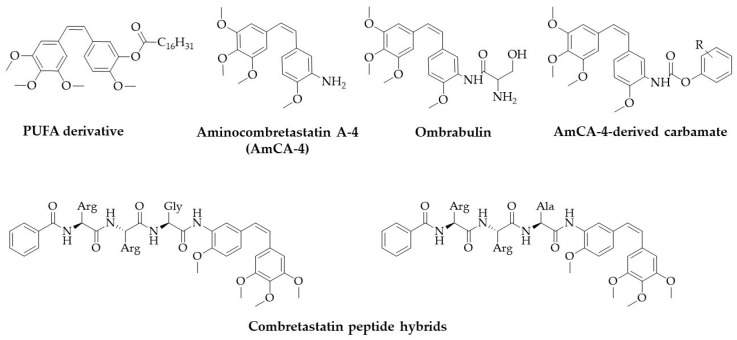
The structure of selected CA-4 derivatives with the modified hydroxyl group [137,138,139].

Ojike et al. proved that polyunsaturated fatty acids (PUFAs) derivatives possess similar activity as CA-4 towards MCF-7 cell line, while all tested compounds displayed a lower inhibitory activity to tubulin compared to the parental drug [136]. Interestingly, Gu et al. also synthesized several prodrugs with fatty chains attached at the 3′-position of the CA-4 B-ring varying in length, such as 6, 10, 14, 16, and 18 carbons (Figure 6) [137]. In this study, the authors combined two strategies: they increased the drug lipophilicity to improve pharmacokinetic properties, such as distribution or biological half-time and used a liposomal formulation as a drug delivery system to ensure the uptake by cells with high biocompatibility and low toxicity. The in vitro experiments showed strong cytotoxicity to MCF-7, S180, and HepG2 cells (with IC_50_ values below < 1 µM). Moreover, in vivo studies showed that the most active compound inhibits cancer cell growth with tumour inhibition rate over 90%. Noteworthy, they observed that the chain length plays a critical role in anticancer activity. CA-4-18-L analogue with the longest carbon chain exerted less cytotoxic effect in vitro, while it was more effective than other analogues [137].

The amino group gives a broad scope for the synthesis of new compounds. One of such group is carbamates, which, compared to reference CA-4 and aminocombretastatin A-4 (AmCA-4), showed increased anti-tumour activity on several cell lines (including HT-29, MCF-7, and HeLa) [138]. To date, it was found that AmCA-4, and Ombrabuline, a serine-derivative from AmCA-4, possess strong cytotoxicity, inhibition of tubulin polymerization, and antivascular activity (Figure 6). By inserting a carbamate group into AmCA-4, the cytotoxic and vascular disrupting activity was highly improved. It should be noted that the most active carbamates are the ones bearing chloro-, bromo-, or methoxy- groups in the *meta* position of the phenyl ring. In turn, Agut et al. reported not only the antiproliferative activity of amide derivatives and their ability to inhibit tubulin, but also the possibility of inhibition of the *VEGF*, human telomerase reverse transcriptase (*hTERT*), or *c-Myc* genes [140].

It is worth emphasizing that amide binding is crucial for the activity of compounds with potential antiviral activity [139]. Richter and colleagues developed a series of amino acid derivatives that incorporate the cleavage site for dengue virus (DENV) protease to activate the tubulin ligand within infected cells (Figure 6) [139]. They showed not only lower toxicity of new analogues but also proved the antiviral activity on DENV and Zika viruses at sub-cytotoxic concentrations [139]. In other studies, the authors designed the prodrugs, which can be cleaved by human carboxylesterase-1 (hCE1). This enzyme is highly expressed in the target cells for DENV, such as immune cells of the macrophage- monocytic lineage (as well as hepatocytes and endothelial cells). Interestingly, new prodrugs that contain the leucine cyclopentyl moiety were hydrolyzed by the hCE1, while phenylglycine cyclopentyl ester combretastatins were inert against hydrolysis. Overall, the antiviral activity of these analogues was similar or lower than that of CA-4 or colchicine [141].

Polyglutamine derivatives (PLG-CA4) studied by Qin et al. showed the potential to induce the polarization of cancer-associated macrophages (TAMs) toward the M2-like phenotype in 4T1 metastatic breast cancer and enhance the activity of T lymphocyte [142]. Moreover, the combination of PLG-CA4 and inhibitor of gamma isoform of phosphoinositide 3-kinase (PI3Kγ) improves the therapeutic effect of NLG919, an inhibitor of immune checkpoint indoleamine 2,3-dioxygenase [142]. In a previous study, Liu et al. demonstrated that PLG-CA4 inhibited the growth of cancer cells in the murine colon C26 tumour model reaching IC_50_ value of 34.4 µg/mL after incubation lasting 48 h [143]. These nanosized polymeric CA-4 prodrugs applied at a dose of 50 mg/kg b.w. (injections were carried out on days 1, 5 and 9 via tail vein after tumours formed in by C26 in Balb/mice reached approximately 100 mm^3^) significantly prolonged retention of such a conjugate in plasma and tumour tissue, and anticancer therapy itself was conducted mainly around the tumour vessels due to low tissue penetration in solid tumours [143].

It would seem that the hydroxyl group’s modifications do not result in derivatives with better activity than combretastatin. However, it should be kept in mind that this drug is poorly water-soluble, and the modifications described above significantly increase the solubility of new derivatives. Modifications of the hydroxyl group also allow the obtaining of compounds with a biological activity other than anti-cancer and innovative forms of drug administration.

### 3.3. Modifications Related to the Methylene Bridge—Change without Cyclization

The literature review shows many possibilities available to researchers synthesizing new combretastatin derivatives: (i) the introduction of an electrophilic agent, (ii) the introduction of aromatic systems, (iii) the introduction of modification via various bonds, e.g., amide scaffold.

The representative of the first approach may be a family of cyanostilbenes, in which an additional nitrile substituent proved to be responsible for excellent antitumour activity [144]. A group of novel *trans*-2-quinolyl-, 3-quinolyl-, and 4-quinolyl cyanostilbene derivatives was synthesized and tested on a panel of 60 human tumour cell lines. Interestingly, 2-and 3-quinolyl analogues containing a 3,4,5-trimethoxyphenyl moiety or a 3,5-dimethoxyphenyl moiety exhibited the most potent growth inhibition with GI_50_ at nanomolar concentrations, while the 4-quinolyl-3,4,5-trimethoxyphenyl and 4-quinolyl-3,5-dimethoxyphenyl analogues were inactive [144].

Of course, it is also possible to directly introduce an aromatic substituent into the combretastatin structure. To combine the anticancer effects of CA-4 and *iso*CA-4 within a single compound, Rasolofonjatova et al. synthesized a family of hybrids that included both the basic skeleton of CA-4 and the one of *iso*CA-4 [145]. However, it seems that such derivatives should be approached with caution because it was found that the most potent analogue has an IC_50_ value of 5 µM and it was less active compared to CA-4 (IC_50_ value of 2 nM) and *iso*CA-4 (IC_50_ value of 2nM) [145].

Based on the literature review, it can be concluded that probably the most popular method of methylene bridge modification is the introduction of a substituent by a chemical bond. The most common is the amide group. Such a strategy was used in the hybrid formation technique. The combretastatin and endoxifen hybrid showed anticancer activity against the estrogen receptor (ER) positive MCF-7 cells. The most active conjugate exhibited nanomolar activity and showed strong competitive ER-binding in ERα (IC_50_ value of 0.9 nM) and ERβ (IC_50_ value of 4.7 nM) [146]. Further research also showed that combining the cyclofenil with CA-4 via amide linking system may serve as a promising selective estrogen receptor modulator (SERM), with an IC_50_ value of 187 nM and binding affinity to ERα (IC_50_ value of 19 nM) and ERβ (IC_50_ value of 229 nM) [147].

Following this strategy, Jadala et al. designed β-carboline-combretastatin carboxamides, which exhibited strong anticancer activity. The most potent compound (Figure 4) showed strong cytotoxic activity with IC_50_ values of 1.01 ± 0.09 µM, 1.51 ± 0.13 µM, 9.97 ± 1.81 µM for A549, DU-145, and HeLa cells, respectively [115]. Moreover, this hybrid arrested A549 cells in the G2/M phase of the cell cycle, induced apoptosis, and generated ROS formation. Interestingly, this compound interfered with the catalytic activity of the topoisomerase-II enzyme and may act as a catalytic inhibitor [115]. The molecular hybridization technique mentioned above was also used to synthesize the CA-4-linked sulfonyl piperazine hybrid [148]. An additional sulphone bridge, used here, was responsible for cytotoxic activity on a panel of cancer cell lines. The most active compounds had the IC_50_ value of 0.36±0.02 µM, 1.75 ± 0.44 µM, 2.16 ± 0.83 µM, 7.05 ± 3.36 µM, 4.08 ± 1.10 µM, 0.92 ± 0.01 µM for A549, MDA- MB-231, MCF-7, B16F10, HCT-15 and HaCaT cell lines, respectively. This compound was further studied using A549, and it was found that it induced apoptosis in a dose-dependent manner and cycle arrest in A549 cells at the G2/M phase. In addition, this compound efficiently inhibited tubulin polymerization with an IC_50_ value of 5.24 ± 0.06 μM. Furthermore, in silico analysis also confirmed that this compound might bind into the combretastatin-binding space on the colchicine binding site of the tubulin [148].

The addition to the aliphatic amide derivatives described above, it is also possible to obtain cyclic amides. O’Boyle et al. synthesized a series of piperazine derivatives, whose biological target is microtubules. Anticancer activity was proved in MCF-7 breast cancer cells [149]. Compounds were tested for tubulin depolymerizing activity and proapoptotic activity. The authors suggest that after increasing the solubility, these compounds may find application in the treatment of triple-negative breast cancer [99,149].

It seems that two fundamental issues should be considered the substitution at the combretastatin methylene bridge: (i) the size of the substituent and (ii) the chemical properties. In the first case, the size of the aliphatic or aromatic scaffold may affect the *cis*/*trans* isomerism of the new derivative during the synthesis process. The planned substituents can be a spatial hindrance, which will reduce synthesis efficiency. In terms of chemical nature, consideration should be given to all polar atoms or substituents that may play a role in the binding process of a new derivative in a biological target.

### 3.4. Modifications Related to the Methylene Bridge—Change with Cyclization

**Figure 7:**
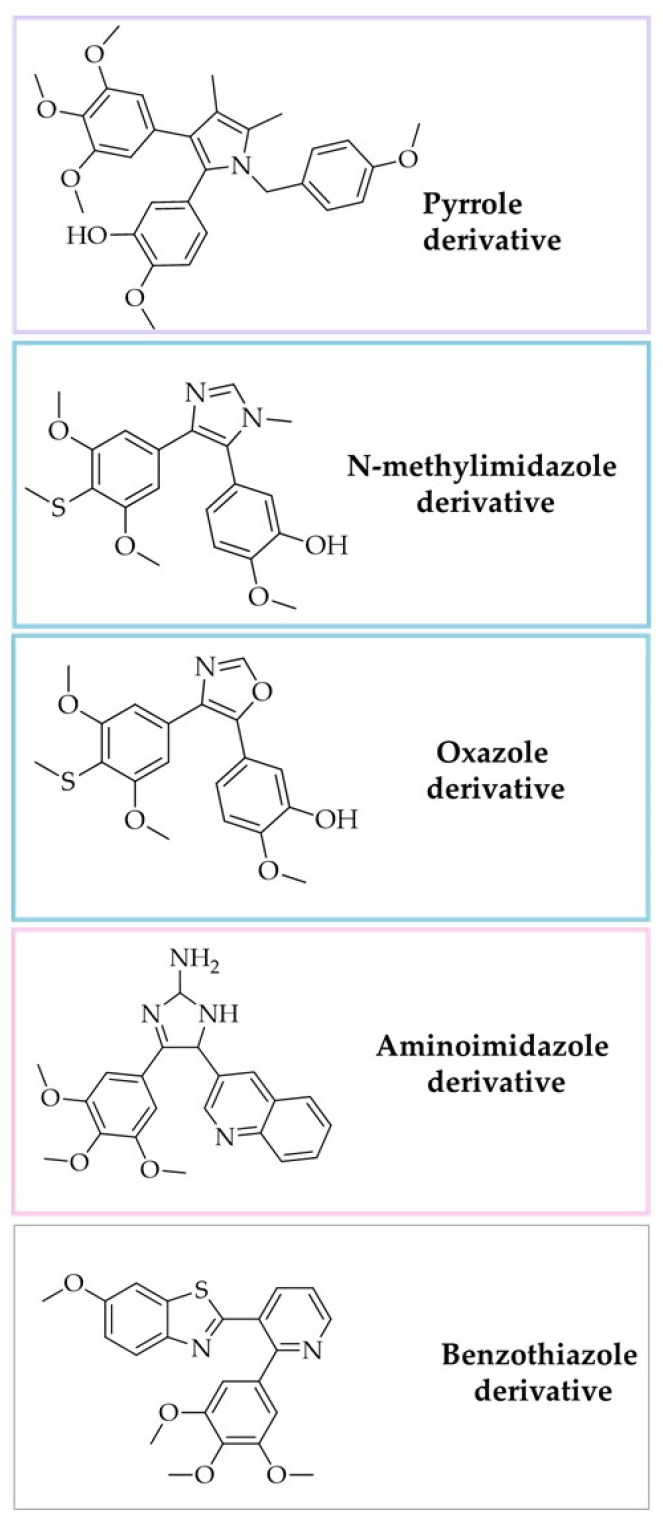
The structure of selected CA-4 derivatives with the modified methylene bridge [150,151,152,153].

One of the strategies applied in the synthesis of combretastatin analogues is the addition of aromatic rings on the methylene bridge. The structures of selected analogues are presented in Figure 7, and their biological activities are presented in Table 2.

One of the chemists’ strategies is to build aromatic rings in such a way that the 3,4,5-trimethoxyphenyl and 2-hydroxy-3-methoxyphenyl, critical for activity, are connected to the methylene bridge. The pyrrole derivative of CA-4 exerted an excellent cytotoxic activity with an IC_50_ value of 70 nM against breast cancer MDA-MB-231 cell line [150] (Figure 7, Table 2). Of course, pyrrole is not the only azaheterocyclic scaffold used to prepare combretastatin analogues. Literature research indicates oxazole, indazole, or thiophene derivatives [151,154,155,156,157]. Stefanski et al. found that N-methylimidazole-bridged CA-4 analogues were significantly less active than their corresponding oxazole analogues (Figure 7, Table 2) [151]. These data suggested that replacement of the oxazole with the N-methylimidazole moiety may be associated with decreased cytotoxic activity [151]. Moreover, the N-methylimidazole derivatives exerted weakly on tubulin polymerization, while oxazole derivatives were more potent inhibitors compared to positive control CA-4 [151].

Aminoimidazole derivatives present another interesting group. Chaudhary and colleagues synthesized a family of 4,5-diaryl-2-aminoimidazole analogues with strong cytotoxic activity and microtubules depolymerization at the nanomolar concentration (Figure 7, Table 2) [152].

The strategy described above can also be used to incorporate organic 6-cyclic bases, such as pyridine [153,158], pyrazoline [159], and pyrimidine [160]. Ashraf et al. replaced the ring B of combretastatin structure with benzimidazole and benzothiazole scaffolds (Figure 7, Table 2) [153]. They found that the methoxy group on C-6 position of benzimidazole and benzothiazole moiety was essential for imparting anticancer activity. Moreover, the presence of trifluoromethyl on C-5 and chlorine atom on C-2 position A and the methoxy group on C-6 position of benzothiazole ensured stronger activity than CA-4 [153]. It is worth noting that, besides anticancer activity, anti-inflammatory and antioxidant activities have also been reported for pyrazoline derivatives [159]. It has also been proven that the mechanism of action lies in the inhibition of antioxidant enzymes that cause elevated ROS levels [160].

Combretastatin derivatives with pyrazole moiety are quite remarkable groups that do not have the crucial functions of phenyl rings attached to the methylene bridge. This group can be illustrated by pyrazole derivatives, which can activate the Ras Homolog Family Member A and Rho-associated protein kinase (RhoA-ROCK) pathway [161]. Brown et al. developed a modular synthetic route to combretastatin analogues based on a pyrazole core through highly regioselective alkyne cycloaddition reactions of sydnones [161]. A close analogue, the derivative of 1,2,3-triazole, also does not have the typical stilbene system but has shown antitumour activity against acute lymphoblastic leukaemia (CEM) and MDA-MB-231 cells [162].

Mustafa et al. synthesized a series of *cis* restricted 1,2,4-triazole analogues of combretastatin A-4 with as promising anticancer drug candidates [163]. The antiproliferative activity of these compounds was tested on hepatocellular carcinoma (HepG2), leukaemia (HL-60), and breast cancer (MCF-7) cell lines. These results showed a substantial ability of the synthesized analogues to inhibit tumour growth. Further studies showed that the most potent analogues showed approximately the same ability to inhibit tubulin polymerization when compared to CA-4 and affinity to the colchicine binding site. The molecular modelling showed several hydrogen bonding and van der Waals interactions with many important amino acids inside the colchicine binding site of tubulin [163]. Interestingly, the authors found that tested compounds less interact with β-tubulin, compared with combretastatin CA-4, probably due to their bulkier nature. The substitution of the ethoxy phenyl ring by halogenated allows one to obtain the component with procaspase-3 activity [163]. Further modification of the molecules, e.g., by cyclization, can lead to indole derivatives [164]. For example, a derivative showed activity against glioblastoma (SNB-75), and renal (UO-31 and CAKI-1) cell lines.

Modifying the combretastatin molecule by adding an aromatic ring in the place of the methylene bridge provides a number of exciting possibilities. Such modifications, depending on the type of ring, offer the possibility of synthesis derivatives with novel biological activities or new anticancer mode of action. However, the final derivative’s solubility in water should always be taken into account, which, in the case of combretastatin, is one of the critical problems.

## 4. Significant Progress of Benzanilides-Small Group with Large Potential

**Figure 8:**
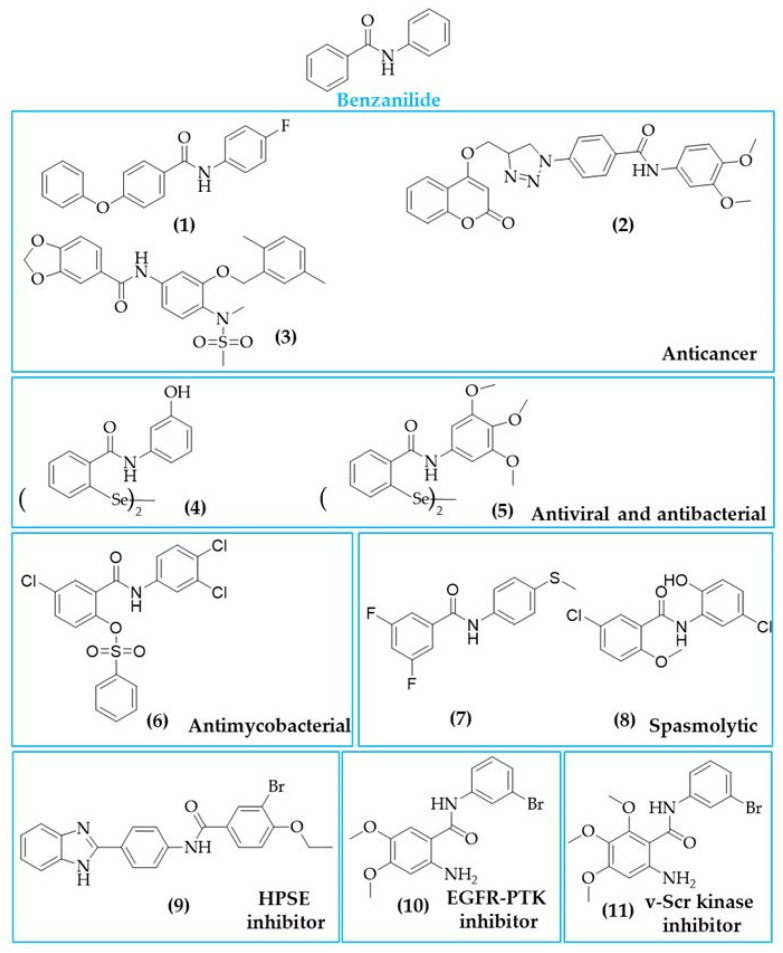
The structures of selected benzanilides with different biological activities. **1**: N-(4-Fluorophenyl)-4-phenoxybenzamide [165]; **2:** N-(3,4-Dimethoxyphenyl)-4-(4-(((2-oxo-2H-chromen-4-yl)oxy)methyl)-1H-1,2,3-triazol-1-yl)benzamide [166]; **3**: N-(3-((2,5-dimethylbenzyl)oxy)-4-(N-methylmethylsulfonamido)phenyl)benzo[d][1,3]dioxole-5-carboxamide [167]; **4**: Bis[2-(2-hydroxyphenylcarbamoyl)]phenyl diselenide [168]; **5**: Bis[2-(3,4,5-trimethoxyphenylcarbamoyl)]phenyl diselenide [168]; **6**: 4-Chloro-2- (3,4-dichlorophenylcarbamoyl)phenyl benzenesulfonate [169]; **7**: N-(4-Methylthiophenyl)-3,5-difluorobenzamide [170]; **8**: N-(2-hydroxy-5-chlorophenyl)-(2-methoxy-5-chloro)-benzamide [171]; **9**: N-(4-(1H-benzo[d]imidazol-2-yl)phenyl)-3-bromo-4-ethoxybenzamide [172]; **10**: 2-amino-N-(3-bromophenyl)-4,5-dimethoxybenzamide [173]; **11**: 6-amino-N-(3-bromophenyl)-2,3,4-trimethoxybenzamide [173]. Abbreviations: ABCG2, ATP Binding Cassette Subfamily G Member 2; DDR1, Discoidin domain receptor 1; EMCV, Encephalomyocarditis virus; HBV, Hepatitis B Virus, HHV, human Herpesvirus 1; HPV, human Papillomaviruse; IRF-1, interferon regulatory factor-1; MMP, matrix metalloproteinase; TRPV1, transient receptor potential vanilloid subfamily member 1; v-Src, Proto-oncogene tyrosine-protein kinase; V1A, Vasopressin receptor 1A; V2, Vasopressin receptor 2.

A modification of the central alkene of the stilbene scaffold, accomplished by replacing the alkene with an amide bond substituted, led to the formation of benzanilide-based compounds. In this chapter, we mainly focused on benzanilide and its close relatives’ thiobenzanilides. Some representative structures for both groups are presented in Figure 8.

Several different activities and molecular targets have been determined for the benzanilide scaffold, and the broad spectrum of activity is presented in Table 3. The chemical beauty of benzanilide remains in structural simplicity and a broad spectrum of possible modifications, which render this structure an attractive chemical starting point for new drug candidates.

**Figure 9:**
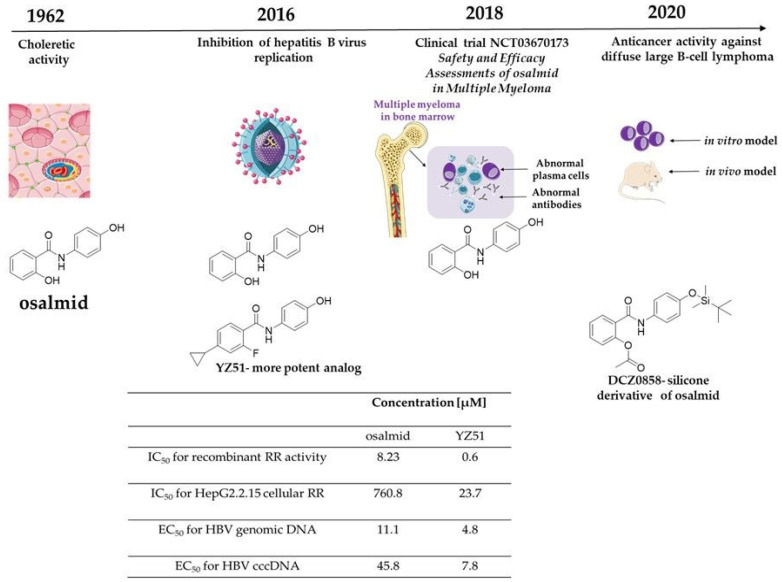
The development of osalmid and its derivatives, as a potent structure in the treatment of certain medical conditions [178,191]. Abbreviations: cccDNA, covalently-closed-circular DNA; DCZ0358 (5-(benzo[d] [1,3]dioxol-5-yl)-3,9,10-trimethoxy-2,3-dihydrooxazolo [2,3-a]isoquinolin- 4-ium chloride); RR, human ribonucleotide reductase; YZ51, 4-cyclopropyl-2-fluoro-N-(4-hydroxyphenyl) benzamide.

For example, 2-hydroxy-N-(4-hydroxyphenyl) benzamide, also known as osalmid (Figure 9) is a medicine used for treating acute and chronic cholecystitis and gallstone disease [178]. Previously, the literature data reported that osalmid is a potential ribonucleotide reductase small subunit M2-targeting compound with potent activity against a lamivudine (3TC)-resistant hepatitis B virus strain in vitro and in vivo [191]. Moreover, the silicone derivative of osalmid (DCZ0858) was found to affected multiple myeloma (MM) cell growth in vitro by dual inhibition of mTORC1/2 and inhibited MM tumour growth in vivo [195]. Lu et al. reported that DCZ0858 exerted an antiproliferative effect on different diffuse large B-cell lymphoma (DLBCL) cell lines with IC_50_ values of 14.4 μM, 9.7 μM, 8.8μM, 11.5 μM, 7.4 μM, 10.1 μM, and 10.7 μM for DB, TMD8, U2932, SUDHL-4, OCI-LY8, OCI-LY1, and NU-DUL-1 cell lines, respectively [178]. Treatment with DCZ0858 was associated with cell cycle block at phase G0/G1, the activation of internal and external apoptotic pathways, and the inhibition of the JAK2/STAT3 pathway [178]. The anticancer effect was confirmed in the OCI-LY8 xenograft model. Administration of DCZ0858 significantly prevented tumour growth by decreasing cell proliferation and inducing apoptosis without causing any damage to important organs [178]. Currently, osalmid is under clinical trial in the treatment of MM (ClinicalTrials.gov).

Besides having a plethora of potential activities, such as spasmolytic, antibacterial, and antiviral, in addition to being drug candidates for pain and osteoarthritis treatment, much attention is paid to the benzanilide scaffold as a base for anticancer agents. Numerous publications have reported anticancer activity of benzanilide analogues, and the introduction of this moiety into the structure can be considered a reasonable option in the design of new drugs. Yang at al. modified the aniline into benzanilide group and identified a potent compound (structure **1** in Figure 8) which inhibited the growth of a panel of cell lines, such as hepatoma (HepG2, Hep3B, PLC/PRF/5, and SMMC-7721), lung (A549), colon (HT-29), cervical (HeLa), and melanoma (A375) cell lines [157]. Interestingly, compound 1 exhibited higher potency in HepG2 (IC_50_ value of 2.57 µM), which is about 6-fold more active than positive control sorafenib. These results demonstrated that compound 1 might be considered as a selective inhibitor of human liver cancer cells. In further studies, compound 1 was able to induce apoptosis with increased expression of the cleaved caspase-3 and p21 [174].

An et al. used the molecular hybridization strategy to link two pharmacophores, coumarin and benzanilide, to increase the anticancer activity [173]. All tested derivatives inhibited MDA-MB-231 cells growth, while the parental compound (4-hydroxycoumarin) was inactive (IC_50_ > 100 µM). The most active analogue (structure 2 in Figure 8) has an IC_50_ value of 0.03 µM and 1.34 µM under hypoxic and normoxic conditions, respectively. It should be emphasized that this compound exerted antiproliferative activity under hypoxic conditions, even better than that of doxorubicin or cisplatin. This compound also had the potential to inhibit carbonic anhydrase IX (CA IX). It should be mentioned that CA IX is an enzyme induced by hypoxia, which plays a role in tumour adaption to an acidic environment, promotes invasiveness, and correlates with therapeutic resistance [196]. Moreover, CA IX expression in non-cancerous tissues is rare and generally confined to epithelia of the stomach, gallbladder, pancreas, and intestine [196]. Thus, CA IX has an emerging potential therapeutic target for anticancer drug development [197].

Analogously, in a study by Zhong and colleagues, this strategy was used to synthesize compound CSUOH0901 (structure 3 in Figure 8), which shares some structural similarity with nimesulide and has a substituted benzamide structure with the electron-donating group at the *para* position. The anticancer activity of CSUOH0901 was confirmed using a panel of 60 cell lines. Studies carried out in the SKBR-3 breast cancer cells have demonstrated potent cytotoxic activity (IC_50_ value of 0.20 μM) and the ability to induce cell cycle arrest at G2/M and apoptosis. The compound was also tested for the acute toxicity to determine the maximum tolerated dose in nude mice, and it was found that it was well tolerated after dosing of 100 mg/kg 200 mg/kg or even 400 mg/kg b.w. Anticancer activity of CSUOH0901 was further confirmed on HT-29 tumour-bearing nude mice. Results showed a decrease in tumour size in mice treated with CSUOH0901 at a dose of 5 mg/kg b.w. administered intraperitoneally five times per week, compared to the control tumour [172].

**Figure 10:**
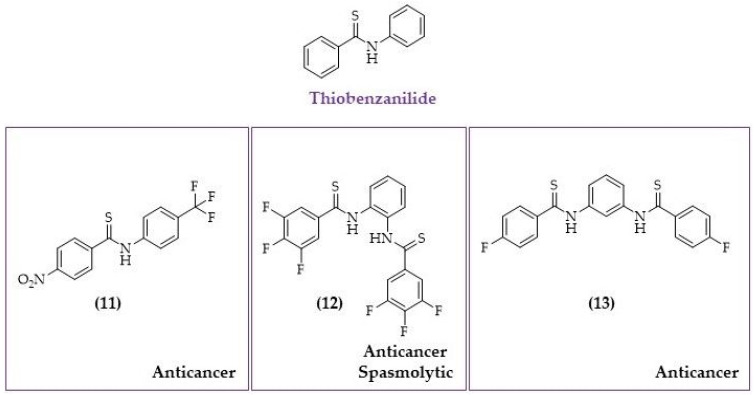
The structures of the most interesting thiobenzanilides with anticancer activity. **11**: N-(4-Trifluoromethyl-phenyl)-4-nitrothiobenzamide [175]; **12**: N,N’-(1,2-phenylene)bis(3,4,5-trifluorobenzothioamide [171,176,177,198]: **13**: N,N’-(1,3-phenylene)bis(4-fluorobenzothioamide) [171].

Hu et al. determined the activity of benzanilides and thiobenzanilides with 4-nitrobenzyl moiety and the differently modified N-aryl fragment using human melanoma A375, leukaemia (K562), and human embryonic kidney (HEK293) cell lines. They found that thiobenzanilides (structure 11 in Figure 10) were more active than their benzanilide counterparts [175]. Their anticancer mechanism was associated with decreased ATP level, inducing oxidative stress via hydrogen peroxide generation, and the induction of caspase-dependent apoptosis [175].

Kucinska et al. tested a huge family of benzanilides and thiobenzanilides and found that a small molecule, referred to as 63T (structure 12 in Figure 10), exerted a potent anticancer activity against lung (A549) and breast (MDA-MB-231, MCF-7) cancer cell lines [171]. Detailed studies performed on A549 and normal lung fibroblast CCD39Lu showed that 63T might serve as a selective anticancer agent with a selectivity index of 2.7 (IC_50_CCD39Lu/IC_50_A549) [176]. Further studies indicated that the selectivity of this compound was caused at least partially by the different responses for ROS in cancer cell line and healthy cells, as well as different metabolic pathways leading to oxidative stress generation. Mechanistic studies showed that 63T selectively induced cancer cell death by activation caspase-independent pathway and inducing oxidative stress. Moreover, 63T affected the expression of several antioxidative and drug-metabolizing enzymes, such as manganese dismutase, catalase, glutathione-S-transferase, and glutathione peroxidase. Moreover, incubation with 63T increased nitric oxide level in both cancer and non-cancerous cell line in a concentration-dependent manner [177].

Stilbene-based compounds may also interact with ERα [199,200] and impact an estrogen metabolism. Interestingly, it was found that thiobenzanilide analogues may act as SERM. Kucinska et al. [171] have reported, that the most potent compound (structure 13 on Figure 10) was highly cytotoxic and selective towards estrogen-dependent MCF-7 cell lines (with an IC_50_ value of 5.07 μM) compared to MDA-MB-231 and healthy breast cells MCF-12A, with IC_50_ values of 100 μM and 91.46 μM, respectively [171]. Docking studies have shown that this compound may interact with the receptor in the same cavity as estradiol. However, it was found that the extra aromatic ring is involved in additional binding interactions with residue W383 (tryptophan at position 383). It was confirmed by using the mutated versions of the ERα receptor that were constructed, and HEK293 cells were transfected with either the wild type or the mutated plasmid. Our results confirmed that interaction with W383 is also required for the binding compound with ERα [171]. Similarly, it was found that W383 also played an important role in stabilizing of the hERα–benzophenone imines complexes and ensured the hydrophobic interaction [201]. Moreover, Landeros-Martinez et al. showed that for tamoxifen analogues, such as amide, carboxyl, and sulfhydryl, the presence of the W383 amino acid of the pocket site might serve as an electron donor [202].

The literature review shows that benzanilide and thiobenzanilide scaffolds are attractive and extremely useful structures. Several mechanistic studies showed that benzanilides offer a different mode of action, which can be modified to create new derivatives with both improved properties and biological activity.

## 5. Conclusions

Scientists from all over the world aim to surpass one another in creating “something new.” Defining a novel drug candidate requires one to show its molecular distinction from a prior structure. Although numerous new structures are designed and published every year, drug development is not limited only by our imagination, but mainly by our knowledge. Thus, the principal strength of every piece of research lies in the knowledge, which brings both positive and negative results. This knowledge is particularly important in terms of new drug discovery, as well as finding the new activity of known structures.

In general, the search for new drugs should be built on a strong base. The concept of a scaffold concerns many aspects at different steps on the drug development path, such as designing libraries of the compounds, the study about the SAR, and defining the specific target. Therefore, the core structures that interact with expected cellular targets are attractive for medicinal chemistry, and such “drug-likeness” scaffolds are a starting point for the generation of specific active agents. This paper aims to present one of the most potent chemical cores: stilbene scaffold. For years, the chemical uniqueness of the stilbene structure has inspired scientists from different fields, such as chemistry, biology, pharmacy, and medicine. An intensive study of the last thirty years showed numerous activities and novel opportunities given by a wide variety of natural compounds as well as synthetic agents.

## Figures and Tables

**Table 1:** The biological activities of isorhapontigenin, pinosylvin, and DMU-212.

Agent	Mechanism		Model	Concentration	Effect	Ref.
	in vitro	in vivo				
**ISORHAPONTIGENIN**	PI3K/Akt↓ CXCL8↓ Il-6↓ Akt↓		Patient-derived AEC		Anti- inflammatory	[52]
	NfκB↓ AP-1↓ nuclear c-Fos↑ c-Jun↓ Akt↓ ROS generation↓ FOXO3A↑		A549			
	Platelet aggregation↓		Human-derived platelets	3.125 μM to 100 μM		[49]
		Haemostasis ↔	C57BL/6 mice	1.85 μM and 6.25 μM		
	NO↓ iNOS↓ PGE2↓ COX2↓ IL-1β↓ MAPK/ERK/p38↓		Rat-derived chondrocytes	10 µM and 20μM		[61]
		FFA↓ PPARγ↑ FaS Fabp4 Glut4	*db*/*db* mice	25 mg/kg b.w.	Anti-diabetic	[26]
	*CEBPα↑* *FaS*↑ *Fabp4↑* *Glut4↑* PPARγ↑		3T3-L1 cells	25 μM		
	↓glucose ↓fatty acid ↓amino acid ↓primary bile acid ↓linoleic acid ↓arachidonic acid ↓pyrimidine metabolism		rats	90 μmol/kg-intravenous dose; 200 μmol/kg 100 μmol/kg-single oral doses; 100 μmol/kg-eight repeated daily oral doses		[50]
			in silico		Antioxidant	[53]
	ROS↓ Nrf2↑		THP-1-XBlue-MD2-CD14 HepG2	2 µM		[55]
		Cyclin D1↓ Sp1↓	T24T mice xenograft	150 mg/kg b.w.	Anticancer	[59]
	Sp1↓ Cyclin D1↓		T24T UMUC3	10 µM		
	XIAP↓	HCT116		20 µM, 40 µM, 60 µM		[58]
	Invasion↓		C57BL/6J mice	150 mg/kg b.w.		[61]
	FOXO1↑		UMUC3	10 µM		
			T24T	20 µM		
	SPHK1/2↓ ROS ↑ c-PARP↑ c-caspase-3↑ cytochrome c↑ c-caspase-9↑ TNFα ↓ IL-6↓ IL-1β↓ ERK↓ Akt↓ Tubulin polymerization↓ Cell cycle arrest		MCF-7	5 µM, 10 µM, 20 µM, 40 µM		[63]
	c-PARP↑ c-caspase-3↑ cytochrome c↑ SPHK1/2↓ Tubulin polymerization↓		MDA-MB-231	20 µM 40 µM		
	c-caspase-3↑ c-PARP-1↑ XIAP↓ Cyclin D1↓ p53↓ *p*-FOXO1↓ *p*-Akt↓ *p*-ERK1/2↓ *p*-EGFR↓ *p*-SP1↓ AR↓		LNCaP CWR22Rv1	20 µM, 50 µM, 100 µM		[64]
		c-capase-3↑ c-PARP↑ XIAP↓ Cyclin D1↓ *p*-FOXO1↓ *p*-Akt↓ *p*-ERK1/2↓ *p*-EGFR↓ AR↓ Ki-67↓	CWR22Rv1 mice xenograft	50 mg/kg b.w.		
	cd44↓ FOXO1↑ c-MYC↓ Sp1↓ USP28↓ miR-4295↑		T24T	20 μM		[61]
	Growth ↓		*S. aureus*	MIC * ranging from 128 to 256 μg/ml	Antimicrobial	[65]
**Pinosylvin**	TRPA1-mediated Ca^2+^ influx↓		HEK293	0.1–100 µM	Anti- inflammatory	[66]
		TRPA1-mediated Ca^2+^ influx ↓ IL-6 ↓	C57BL/6N mice	10 mg/kg b.w.		
	PI3K/ Akt↓ NO↓ IL-6↓ MCP1↓		J774	1–30 µM		[67]
		IL-6↓ MCP1↓	mice	30 mg/kg b.w.		
	IL-6↓ IL-1β↓ IL-17 aggrecan expression↑		osteoarthritis chondrocytes	100 µM		[68]
	NF-κB↓		T/C28a2			
	iNOS↓ NO↓ MCP-1↓ IL-6↓		J774	3 to 100 μg/mL (*P. sylvestris* extract)		[69]
		inflammation↓	mice	100 mg/kg b.w.		
	NF-κB↓		HEK293	100 µM	Antioxidant	
	GLUT4↑ *p*-AMPK↑ SIRT1 activity↑	Rat L6 myoblasts		20 µM, 60 µM, 100 µM	Anti-diabetic	[70]

	Adipocytes proliferation↓ PPARγ↓ C/EBPα↓ TNFα ↓/ IL-6↓		Mouse 3T3-L1 preadipocyte	20–60 μM	Adipogenesis inhibition/ anti- inflammatory	[71]
	MMP-2↓ TIMP-2↑ ERK1/2↓		SCC- 9HSC-3 SAS	20 µM, 40 µM 80 μM	Anticancer	[72]
	caspase-3↑ LC3-II↑ p62↓ AMPKα1↓ autophagy/ apoptosis↑		THP-1 U937	0–100 μM		[73]
**DMU-212**	*IL8*↑ *EGR1*↑ *ERRFI1*↑ *TRPC4*↑ *BIRC3*↑ *CYP1B1↓* *MVK*↓		HUVECs	20 µM for 6 h	Diverse range of genes regulation	[74]
	AhR↑		HepG2	10 µM to 50 µM		[75]
	Bcl-2↑ caspase-3 and -9↑		HUVECs	5–80 μM	Apoptosis	[76]
	VEGF- induced migration↓ VEGFR2 pathway↓				Angiogenesis	
		VEGF- stimulated angiogenesis↓	mice chick eggs			
	p21↑ p53↑ cyclin B1↑ caspase-3 and -9↑ Bax↑ Bcl-2↓ ERK1/2↑ MEK1/2↓		A375, MeWo M5 Bro	0.312–540 µM	Anticancer	[77]
		GPx-1↓ CAT↓ GR↓ SOD-2↓ GST↓ *apaf-1* *stat-1* *pten*↓ caspase-9 mRNA↓ Socs-2↑ Tnfsf10↓ Tnfrsf1a↓ Tnfsf1↑	Wistar rats	50 mg/kg by gavage 20 or 50 mg/kg b.w. twice/week for 16 weeks	Antioxidant system	[78]

Abbreviations: AP-1- activator protein 1; Apaf-1, Apoptotic protease activating factor 1; Bax, apoptosis regulator protein; Bcl-2, B-cell lymphoma 2; BIRC3-baculoviral IAP Repeat Containing 3; c-Jun, Jun proto-oncogene, AP-1 transcription factor subunit; c-MYC-regulator gene that code for transcription factors; cd44-glycoprotein antigen in a cell-surface; c-Fos, Proto-oncogene c-Fos; CXCL8; chemokine (C-X-C motif) ligand 8; EGR1-early growth response protein 1; ERRFI1-ERBB receptor feedback inhibitor 1; Fabp4-fatty acid-binding protein 4; FFA-free fatty acid; FAS-fatty acid synthase; IL, interleukin; IL-1β-interleukin1β; Ki-67, nuclear protein; miR-4295-Hsa-microRNA-4295; MVK- mevalonate kinase; Nf-κB- nuclear factor kappa-light-chain-enhancer of activated B cells; NPGE2-prostaglandin E2; p38-mitogen-activated protein kinases; p21-cyclin-dependent kinase inhibitor 1; p62-the ubiquitin-binding protein; Pten, phosphatase and tensin homolog deleted on chromosome ten; Socs-2-suppressor of cytokine signaling 2; Stat-1, signal transducer and activator of transcription 1; TIMP-2-tissue inhibitor of metalloproteinases 2; Tnfsf -tumor necrosis factor (ligand) superfamily; TRPC4- the short transient receptor potential channel 4.

**Table 2:** The summary of biological activity of selected CA-4 derivatives with modified the methylene bridge.

Derivative	Cytotoxicity				Anti-Tubulin Activity	Ref.
	IC_50_ [µM]				IC_50_ [µM]	
	Cell line					
	MDA-MB-231	MCF-7	A549	HeLa		
Pyrolle	0.07 ± 0.02				nd *	[150]
Positive control CA-4	0.03 ± 0.0001				nd *	
N-methylimidazole	0.39 ± 0.28	1.63 ± 0.27	>20	0.39 ± 0.02	6.67	[151]
Oxazole	0.71 ± 0.16	0.45 ± 0.14	>20	0.009 ± 0.002	1.05	
Positive control CA-4	0.56 ± 0.08	0.17 ± 0.04	>20	0.11 ± 0.06	2.72	
Aminoimidazole	0.096 ± 0.013	0.003 ± 0.002		0.010 ± 0.001	1.6	[152]
Positive control CA-4	0.331 ± 0.032	0.018 ± 0.002		0.025 ± 0.002	1.7	
Benzothiazole			0.13 ± 0.01	0.06 ± 0.001	2.01	[153]
Positive control CA-4			0.06 ± 0.003	0.06 ± 0.002	1.87	

* nd- no data.

**Table 3:** The molecular targets and activities of benzanilide-based compounds.

Target/Activity		Mode of Action	Ref.
ABCG2		inhibition	[174,175]
Bacteria	*Escherichia coli*	inhibition	[168,176]
	*Enterococcus* *faecalis*		[168] Structure 4 and 5
	*Enterococcus hirae*		
	*Staphylococcus aureus*		
	*Staphylococcus epidermidis*		
	*Mycobacterium* *tuberculosis*		[169] Structure 6
	*Mycobacterium* *avium*		
	*Mycobacterium* *kansasii*		
Calcium channel		inhibition	[177]
Carbonic anhydrase IX		inhibition	[153] Structure 2
Cancer cells	**Breast**		
	MDA-MB-231	growth inhibition	[178,179,180] Structure 2 and 3
	MCF-7		[178]
	SKBR-3		[179] Structure 3
	**Cervical**		
	HeLa		[165] Structure 1
	**Colon**		
	HT-29		[165,167] Structure 1 and 3
	**Hepatoma**		
	HepG2		[165] Structure 1
	Hep3B		[165] Structure 1
	PLC/PRF/5		
	SMMC-7721		
	**Leukemia**		
	K562		[165,167,179] Structure 1 and 3
	**Lung**		
	A549		[165,167,178,180,181] Structure 1 and 3
	**Lymphoma**		
	DB		[182]
	TMD8		
	U2932		
	SUDHL-4		
	OCI-LY1		
	OCI-LY8		
	NU-DUL-1		
	**Melanoma**		
	A375		[165,179] Structure 1
DDR1		inhibition	[183]
EGFR		inhibition	[173] Structure 10
Estrogen receptor		estrogenic activity	[178]
Histone deacetylase 1		inhibition	[184,185]
Histone deacetylase 2		inhibition	
Histone deacetylase 3		inhibition	
Histone deacetylase 11		inhibition	
HPSE		inhibition	[172] Structure 9
IRF-1		inhibition	[186]
MMP-2		inhibition	[187]
MMP-9			
MMP-13		inhibition	[186]
Potassium channel		activation	[171,188] Structure 8
Quinone reductase-2		inhibition	[189]
TRPV1		inhibition	[190]
v-Src		inhibition	[173] Structure 11
Vasopressin V1A V2		inhibition	[191]
Viruses	HHV-1	inhibition	[168] Structure 4 and 5
	EMCV		
	HBV		[192]
	HPV		[193]
	HIV-1		[194]

Abbreviations: ABCG2, ATP Binding Cassette Subfamily G Member 2; DDR1, Discoidin domain receptor 1; EMCV, Encephalomyocarditis virus; HBV, Hepatitis B Virus, HHV, human Herpesvirus 1; HPV, human Papillomaviruse; IRF-1, interferon regulatory factor-1; MMP, matrix metalloproteinase; TRPV1, transient receptor potential vanilloid subfamily member 1; v-Src, Proto-oncogene tyrosine-protein kinase; V1A, Vasopressin receptor 1A; V2, Vasopressin receptor 2.
